# Mannan-Containing Polymers from Hadal Bacterium *Psychrobacter pulmonis*: Preparation, Structural Analysis, Immunological Activity and Antitumor Effects

**DOI:** 10.3390/md23080326

**Published:** 2025-08-12

**Authors:** Mingxing Qi, Shuqiang Yan, Yukun Cui, Yanan Huang, Yang Liu, Wenhui Wu, Xi Yu, Peipei Wang

**Affiliations:** 1 College of Food Science and Technology, Shanghai Ocean University, Shanghai 201306, China; 18616521497@163.com (M.Q.); sq_yan2020@163.com (S.Y.); 18638611062@163.com (Y.H.); whwu@shou.edu.cn (W.W.); 2 College of Oceanography and Ecological Science, Shanghai Ocean University, Shanghai 201306, China; d240300081@st.shou.edu.cn; 3 College of Marine Living Resource Sciences and Management, Shanghai Ocean University, Shanghai 201306, China; y_liu@shou.edu.cn; 4 Marine Biomedical Science and Technology Innovation Platform of Lin-Gang Special Area, Shanghai 201306, China

**Keywords:** hadal bacterium, extracellular polysaccharide, mannan-containing polymers, structural characterization, immunological activity, antitumor effects

## Abstract

Microbial exopolysaccharides from extreme environments are increasingly becoming valuable candidates for drug development. In this study, four fractions named XL-1, XMRS-1, XL-1-D, and XMRS-1-D were isolated and purified from the hadal bacterium *Psychrobacter pulmonis* by column chromatography. The structural features of these fractions were characterized by molecular weight, monosaccharide composition, Fourier transform infrared (FTIR) spectrum, amino acid analysis and NMR. The results showed that XL-1 and XMRS-1 were mainly composed of mannose, glucose, and glucosamine, while XL-1-D and XMRS-1-D were mainly composed of mannose. In vitro bioactivity assays demonstrated that all four fractions significantly enhanced RAW264.7 macrophage proliferation and phagocytosis, stimulated nitric oxide (NO) and reactive oxygen species (ROS) production, and induced the secretion of interleukin-6 (IL-6), tumor necrosis factor-α (TNF-α), and the expression of inducible nitric oxide synthase (iNOS) mRNA. Moreover, plate cloning tests, cell scratch tests, and apoptosis assays, along with RT-qPCR analysis, demonstrated that the four fractions significantly inhibited A549 cells’ proliferation. Specifically, XMRS-1 and XMRS-1-D upregulated Bax, Caspase-3, Caspase-8, and Caspase-9, while downregulating Bcl-2, suggesting transcriptional activation of apoptosis-related pathways. These results offered a reference for the further development and utilization of this hadal bacterium in the future.

## 1. Introduction

Exopolysaccharides (EPS) produced by marine microorganisms, especially extreme microorganisms, are becoming the focus of increasing attention due to their complex structure, biodegradability, biocompatibility, and bioactivity [1,2,3]. EPS are usually a high molecular weight carbohydrate polymer secreted by microorganisms during growth [4,5,6,7,8]. Because of their high efficiency and renewable raw material characteristics, they have become an important biomaterial in food, medicine, cosmetics, and the environment [9,10,11,12]. The hadal biosphere refers to the trench area with a water depth of more than 6000 m, accounting for 45% of the vertical depth of the ocean. This habitat is characterized by high hydrostatic pressure (>60 MPa), low temperature (<4 °C), and a relatively isolated environment [13,14,15]. Microorganisms in hadal environments have unique adaptations to cope with conditions of high pressure, low temperature, high salinity, and complete darkness [16,17,18]. Novel compounds can often be found in such environments, due to the special living environment. Among them, EPS are an important factor for the survival of hadal microorganisms in extreme environments [1,19,20]. Their potential applications in the pharmaceutical, cosmetic, and food industries, environmental remediation, and astrobiology have attracted widespread interest [21,22,23,24,25]. For example, Novel EPS named as AVP-214-1 from Mariana Trench fungus *Aspergillus versicolor* SCAU214 exhibits immunostimulatory activity, highlighting potential for biotechnological applications of deep-sea polysaccharides [26]. Acidic EPS from the marine actinomycete *Streptomyces* AMG31 shows anti-inflammatory, antioxidant, and anti-Alzheimer’s disease (via BChE inhibition), plus antibacterial and antibiofilm activities against various pathogens [27]. Alternan EPS from *Leuconostoc citreum* SFL-2-8, featuring a nanostructure and film-forming properties, is effective for food coating preservation [28]. These all indicate that EPS produced by microorganisms in extreme environments demonstrate tremendous potential for development and application.

Some evidence shows that the monosaccharide composition, high molecular weight, hydrophobicity, and multi-charge properties of marine EPS are closely related to its low-temperature protection, water retention capacity, and good thermal stability, which are essential for the survival of psychrophiles, halophiles, and thermophiles [21,29,30]. At present, there are few systematic studies on microbial EPS in the hadal environment. These EPS from extreme environments are considered to be important candidates for the development of new drugs and high-end biomedical materials in the future due to their high stability, good biocompatibility, and potential unique biological activities [31,32]. Therefore, the study of microbial EPS in the hadal environment is particularly urgent and has important scientific significance and application prospects. In-depth exploration of EPS from this special extreme environment is of great significance for the discovery of new polysaccharide resources with excellent performance and potential for medical applications. However, little is known about the chemical structural diversity, precise physicochemical properties, and specific pharmacological activities of hadal EPS. Therefore, strengthening the systematic exploration, fine structural characterization, and in-depth evaluation of the biological functions of hadal microbial EPS is expected to screen out new polysaccharide molecules with unique structures and significant efficacy, and open up new avenues for the development of innovative drugs and high-performance biomedical materials with independent intellectual property rights, which has important scientific value and huge potential economic benefits.

*Psychrobacter*, a genus of Gram-negative, psycho-tolerant or psychrophilic, aerobic or facultatively anaerobic coccobacilli, was first described by Juni and Heym in 1986. Noted for thriving in cold environments like marine sediments, Antarctic sea ice, and refrigerated foods, it has garnered research interest for its ecological roles, genomic adaptations, and biotechnological potential [5,33,34]. At present, there have been many studies on this genus. Kokoulin and colleagues identified a capsular polysaccharide from the marine bacterium *Psychrobacter marincola* KMM 277T that exhibits significant antitumor properties, establishing it as a candidate for future mechanistic studies [35]. Kondakova and colleagues isolated and structurally characterized a novel acidic polysaccharide from the Siberian cryopeg isolate *Psychrobacter maritimus* 3pS, determining its repeating unit by means of chemical analysis and NMR spectroscopy [36]. Casillo and colleagues isolated and structurally characterized a mannan exopolysaccharide from the permafrost-derived bacterium *Psychrobacter arcticus* strain 273-4, elucidating its chemical structure and conformational properties through comprehensive analytical approaches [37]. While prior investigations have documented analogous exopolysaccharide constituents, studies focusing on *Psychrobacter pulmonis* remain scarce in the literature. In this study, four fractions, XL-1, XL-1-D, XMRS-1, and XMRS-1-D, were firstly isolated and purified from hadal bacterium *Psychrobacter pulmonis.* The structural character of XL-1, XL-1-D, XMRS-1, and XMRS-1-D was evaluated by chemical composition, molecular weight, monosaccharide composition, infrared spectroscopy, and NMR analysis. Subsequent in vitro bioactivity evaluation showed that the fractions had significant immunomodulatory properties and effective anti-tumor activity. These results comprehensively describe the characteristics of XL-1, XL-1-D, XMRS-1, and XMRS-1-D from hadal bacterium *Psychrobacter pulmonis* and provide a foundation for their high-value utilization and development.

## 2. Result

### 2.1. Extraction, Separation and Purification of XL-1, XL-1-D, XMRS-1, and XMRS-1-D

**Figure 1:**
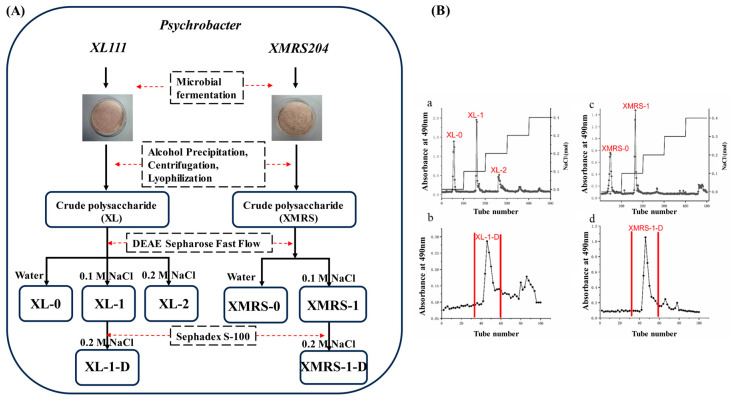
Extraction, separation and purification of XL-1, XL-1-D, XMRS-1, and XMRS-1-D from hadal bacteria (**A**) Flow chart of extraction, purification and preparation of XL-1, XL-1-D, XMRS-1, and XMRS-1-D from microorganisms; (**B**) (**a**) Elution curve of crude fractions of strain XL111 after purification by DEAE-cellulose column chromatography; (**b**) Elution curve of component XL-1 (the location of the characteristic peak in (**b**)) Sephadex S-100 gel filtration chromatography; (**c**) Elution curve of crude fractions of strain XMRS204 after purification by DEAE-cellulose column chromatography; (**d**) Elution curve of component XMRS-1 (the location of the characteristic peak in (**c**) after purification by Sephadex S-100 gel filtration chromatography).

The extraction, purification and preparation flow chart for XL-1, XL-1-D, XMRS-1, and XMRS-1-D is shown in Figure 1A. After alcohol precipitation, centrifugation, lyophilization and protein removement, two crude fractions named XL and XMRS were obtained with yield of 624 mg/L and 1924 mg/L from strain XL111 and strain XMRS204, respectively. These two crude extracellular fractions were further purified by two-step chromatography. Firstly, two main fractions named XL-1 (42.1%) and XMRS-1 (13.3%) were purified from these two crude extracellular fractions by DEAE column separation, respectively. Subsequently, XL-1 and XMRS-1 were purified by Sephadex S-100 gel filtration chromatography to obtain single symmetrical peaks, named XL-1-D (37%) and XMRS-1-D (25%), respectively (Figure 1B).

### 2.2. Structural Characteristics of XL-1, XL-1-D, XMRS-1, and XMRS-1-D

**Figure 2:**
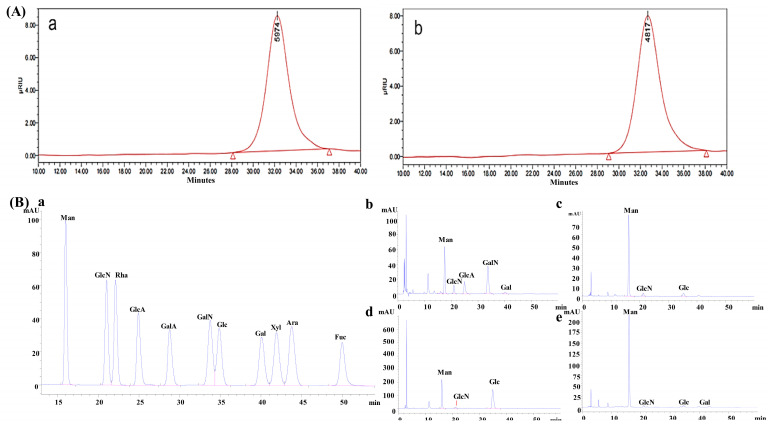
Results of molecular weight and monosaccharide composition of XL-1, XL-1-D, XMRS-1, and XMRS-1-D. (**A**) Molecular weight of XL-1-D and XMRS-1-D ((**a**) Molecular weight of XL-1-D; (**b**) Molecular weight of XMRS-1-D diagram); (**B**) Determination of monosaccharide composition of XL-1, XL-1-D, XMRS-1, and XMRS-1-D ((**a**): monosaccharide standards mixed label; (**b**): XL-1; (**c**): XL-1-D; (**d**): XMRS-1; (**e**): XMRS-1-D).

The total sugar, protein, and uronic acid contents of XL-1, XL-1-D, XMRS-1, and XMRS-1-D were determined. The results in Table 1 show that the total sugar contents of XL-1, XL-1-D, XMRS-1, and XMRS-1-D were 43.56%, 66.82%, 37.54%, and 49.80%, respectively. Their uronic acid contents were 3.65%, 5.14%, 5.27%, and 5.60%, while the protein contents were 17.31%, 10.02%, 13.99%, and 12.30%, respectively. The molecular weights of XL-1-D and XMRS-1-D were determined to be 5.974 kDa and 4.817 kDa by HPGPC method, respectively (Figure 2A). The monosaccharide composition results (Figure 2B) showed that XL-1 was primarily composed of mannose, glucosamine, glucuronic acid, galactosamine, and galactose, with a molar ratio of 35.5:7.3:14.7:36.8:2.8, respectively. XMRS-1 was mainly composed of mannose, glucosamine, and glucose, in a molar ratio of 56.4:2.0:41.6. XL-1-D consisted of mannose, glucosamine, and glucose, with a molar ratio of 89.4:4.4:6.2. XMRS-1-D was composed of mannose, glucosamine, glucose, and galactose, in a molar ratio of 93.3:2.0:3.7:1.0. These results showed, after purification by gel column chromatography, that the monosaccharide compositions of XL-1, XL-1-D, XMRS-1, and XMRS-1-D from both bacterial strains significantly changed compared with those before purification, with fewer types of monosaccharides, and mainly composed of mannose. Here, two fractions from these hadal bacterial strains were obtained.

To eliminate potential interference from mannan in the sterile yeast culture medium in the experimental results, the same ethanol precipitation and deproteinization procedures of the culture medium were performed at 4 °C, albeit with low yield. Additionally, a study showed that one type of mannan was also isolated from the permafrost-derived bacterium *Psychrobacter arcticus* strain 273-4, with ethanol precipitation combined with gel purification protocols [37].

#### 2.2.1. Infrared Spectroscopic Characterization of XL-1, XL-1-D, XMRS-1, and XMRS-1-D

Fourier transform infrared (FTIR) spectroscopy was performed to characterize the chemical structures of XL-1, XL-1-D, XMRS-1, and XMRS-1-D. These fractions showed similar FTIR characteristics. For example, peaks around 3284–3288 cm^−1^ belonged to O-H stretching vibration, while signals around 2927–2928 cm^−1^ were assigned to C-H stretching vibration. The signals around 1017–1018 cm^−1^ belonged to C-O-C stretching vibration. Besides, peaks were observed at 842–845 cm^−1^, usually indicating the presence of α-pyran ring. These signals all belong to the typical infrared spectra of polysaccharides, preliminarily verifying the polysaccharide properties of the four fractions. Their structural features need to be further determined by methylation and NMR analysis.

#### 2.2.2. Morphological Characterization of XL-1, XL-1-D, XMRS-1, and XMRS-1-D

**Figure 3:**
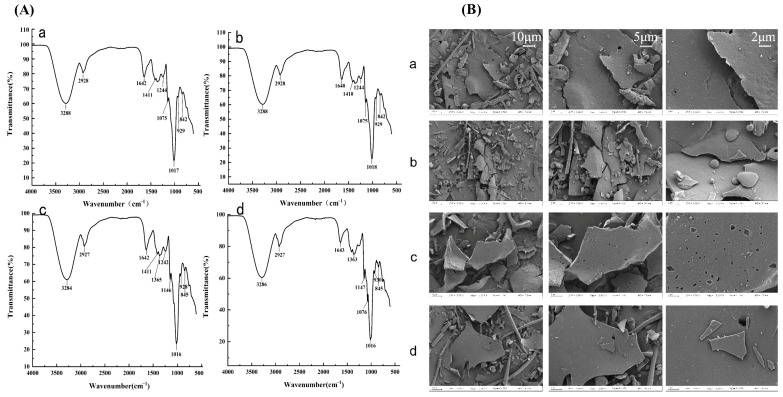
IR of XL-1, XL-1-D, XMRS-1, and XMRS-1-D. (**A**): Infrared spectra of four XL-1, XL-1-D, XMRS-1, and XMRS-1-D ((**a**): XL-1; (**b**): XL-1-D; (**c**): XMRS-1; (**d**): XMRS-1-D); (**B**): SEM of XL-1, XL-1-D, XMRS-1, and XMRS-1-D ((**a**): XL-1; (**b**): XL-1-D; (**c**): XMRS-1; (**d**): XMRS-1-D).

Scanning electron microscopy (SEM) was used to observe the microstructures of XL-1, XL-1-D, XMRS-1, and XMRS-1-D and explore the effects of extraction and purification on their surface morphology (Figure 3B). SEM images of the four fractions exhibited similar lamellar structural characteristics. At higher magnifications, the porous structures on the fractions’ surfaces became more clearly visible. However, due to the limited resolution of scanning electron microscopy, molecular-level structural details could not be observed. Therefore, additional analytical methods are required for further characterization of these four fractions.

#### 2.2.3. Amino Acid Composition of XL-1-D and XMRS-1-D

To determine the amino acid composition of the proteinaceous components potentially associated with these purified fractions, XL-1-D and XMRS-1-D were analyzed. The amino acid profiles, expressed as residues per 1000 total amino acid residues, are detailed in Table 2. Amino acid analysis revealed glycine (Gly) as the predominant amino acid in both purified fractions preparations, XL-1-D and XMRS-1-D. Proline (Pro) and alanine (Ala) were the next most abundant residues. The XL-1-D preparation contained the following residues: Gly: 389.13 ± 10.20, Pro: 137.88 ± 3.65, and Ala: 123.62 ± 3.34. A similar structural result was seen for XMRS-1-D, with (remnants/1000 remnants): Gly: 383.70 ± 1.09, Pro: 136.88 ± 3.65, and Ala: 124.58 ± 1.39.

#### 2.2.4. NMR Analysis of XL-1-D and XMRS-1-D

**Figure 4:**
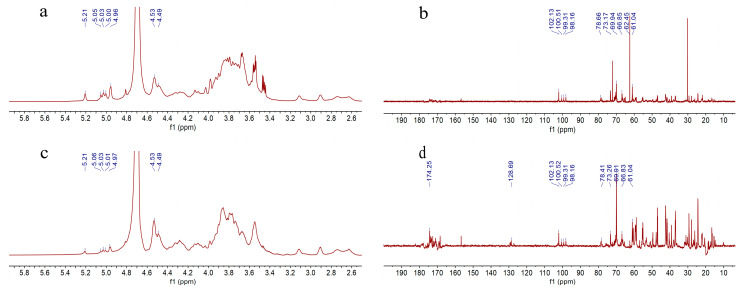
The ^1^H NMR and ^13^C NMR spectra of the two purified fractions XL-1-D and XMRS-1-D. ((**a**), the ^1^H NMR of XL-1-D; (**b**), the ^13^C NMR of XL-1-D; (**c**), the ^1^H NMR of XMRS-1-D; (**d**), the ^13^C NMR of XMRS-1-D).

In order to further standardize the structural characteristics of XL-1, XL-1-D, XMRS-1, and XMRS-1-D, the ^1^H NMR and ^13^C NMR spectra of these two purified fractions XL-1-D and XMRS-1-D were obtained and analyzed, as shown in Figure 4. According to the existing references regarding NMR analysis of mannans and monosaccharides compositions results, the signals of anomeric carbon and hydrogen of mannoses could be initially attributed [27,37,38]. In the ^1^H NMR spectra of XL-1-D and XMRS-1-D, the anomeric hydrogen signals at 5.21 ppm could be deduced to H1 of 2-Manp. The signals around 5.00 ppm were assigned to H1 of 2,6-Manp, while the peaks around 4.5 ppm belonged to H1 of 6-Manp. In the ^13^C NMR spectra of XL-1-D and XMRS-1-D, the signals at 102.1 ppm, 100.5 ppm, 99.3 ppm, and 98.2 ppm were deduced to C1 of 2-Manp, 6-Manp, and 2,6-Manp, respectively. The signals around 61.0 ppm could be assigned to unsubstituted C6 of mannose units, while the signals present at 66.8 ppm belonged to 6-substituted mannose units, since C-6 of these residues were downfield. These data supported the hypothesis of a sugar backbone consisting of α-(1→6)-linked mannopyranose units partially branched at C-2. Besides, according to the literature [39], the signal around 5.34 ppm was attributable to the phosphorylated mannose residues. This suggested that these two fractions did not have phosphorylation substitution, since there was no absorption peak at this position. Although both XL-1-D and XMRS-1-D had very similar chemical shifts for the anomeric hydrogen and carbon, the signals in the H2-H6 region and the C2-C6 region are quite different, partly due to the different proportions of substitution. Furthermore, since both types of polysaccharides are combined with proteins, the presence of different structural amino acids to some extent increased the complexity of the ^1^H NMR and ^13^C NMR spectra of XL-1-D and XMRS-1-D and made it impossible to completely attribute this to all carbon and hydrogen signal shifts.

### 2.3. Immunomodulatory Effects of XL-1, XL-1-D, XMRS-1, and XMRS-1-D

#### 2.3.1. Effect of XL-1, XL-1-D, XMRS-1, and XMRS-1-D on the Viability and Proliferation of RAW264.7 Cells

**Figure 5:**
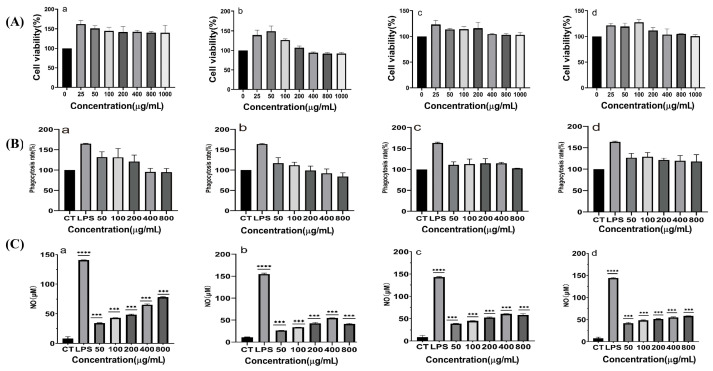
(**A**) Effects of different concentrations of XL-1, XL-1-D, XMRS-1, and XMRS-1-D on the viability of RAW264.7 cells ((**a**): XL-1; (**b**): XL-1-D; (**c**): XMRS-1; (**d**): XMRS-1-D); (**B**) Effects of different concentrations of XL-1, XL-1-D, XMRS-1, and XMRS-1-D on phagocytic activity of RAW264.7 cells ((**a**): XL-1; (**b**): XL-1-D; (**c**): XMRS-1; (**d**): XMRS-1-D); (**C**) Effects of different concentrations of XL-1, XL-1-D, XMRS-1, and XMRS-1-D on NO secretion in RAW264.7 cells ((**a**): XL-1; (**b**): XL-1-D; (**c**): XMRS-1; (**d**): XMRS-1-D). (All data are expressed as mean ± standard deviation (mean ± SD). Differences between groups and the blank control were analyzed using one-way analysis of variance (ANOVA). Significance levels were defined as follows: *** *p* < 0.001 (extremely significant), and **** *p* < 0.0001 (very highly significant)).

RAW264.7 cells were co-cultured with various concentrations of XL-1, XL-1-D, XMRS-1, and XMRS-1-D for 48 h. Cell proliferation was assessed using the CCK-8 method, and the relative proliferation rate was calculated with the untreated control group as a reference (Figure 5A). XL-1 significantly promoted RAW264.7 cell proliferation in the concentration range of 25 to 1000 µg/mL. XL-1-D promoted proliferation between 25 and 200 µg/mL. In the range of 400 to 1000 µg/mL, cell viability was lower than 100% but remained above 80%, indicating no cytotoxicity. Both XMRS-1 and XMRS-1-D promoted RAW264.7 cell proliferation across the concentration range of 25 to 1000 µg/mL without exhibiting cytotoxicity.

#### 2.3.2. Effect of XL-1, XL-1-D, XMRS-1, and XMRS-1-D on Macrophage Phagocytic Function

The phagocytic activity of RAW264.7 cells was evaluated (Figure 5B). XL-1 and XL-1-D significantly increased the phagocytic rate of RAW264.7 cells at concentrations from 50 to 200 µg/mL. However, at concentrations exceeding 400 µg/mL, the phagocytic rate was inhibited. XMRS-1 enhanced the phagocytic rate between 50 and 400 µg/mL, with inhibition observed at 800 µg/mL. XMRS-1-D promoted phagocytic activity in the concentration range of 50 to 800 µg/mL.

#### 2.3.3. Effect of These Fractions on Nitric Oxide (NO) Production by Macrophages

The Griess method was used to indirectly determine NO content by measuring nitrite levels in the culture supernatant, thereby evaluating the effects of XL-1, XL-1-D, XMRS-1, and XMRS-1-D on NO release from RAW264.7 macrophages after 48 h of treatment (Figure 5C). XL-1 significantly and concentration-dependently promoted NO release between 50 and 800 µg/mL (*p* < 0.05). XL-1-D effectively enhanced NO release from 50–400 µg/mL; at 800 µg/mL, the stimulatory effect plateaued, consistent with cell viability results. Both XMRS-1 and XMRS-1-D demonstrated a continuous promotion of NO release in the 50–800 µg/mL range. The positive control group (1 µg/mL lipopolysaccharide, LPS (Sigma-Aldrich)) significantly induced NO release (*p* < 0.01). These findings suggest that all four fractions can enhance macrophage immune activity by increasing NO release, with XL-1 showing a particularly notable effect.

#### 2.3.4. Effect of XL-1, XL-1-D, XMRS-1, and XMRS-1-D on Reactive Oxygen Species (ROS) Production by Macrophages

**Figure 6:**
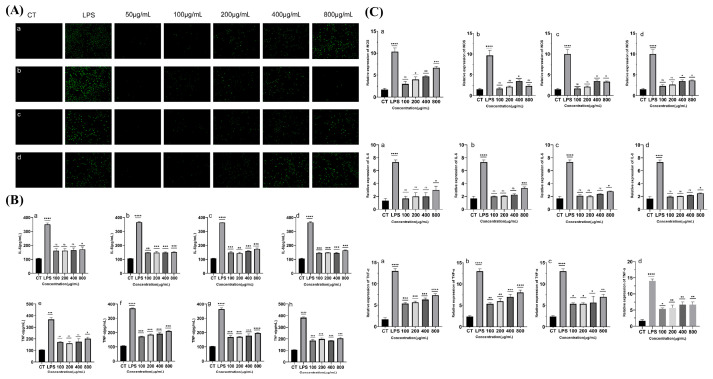
(**A**) Effects of different concentrations of XL-1, XL-1-D, XMRS-1, and XMRS-1-D on ROS secretion in RAW264.7 cells ((**a**): XL-1; (**b**): XL-1-D; (**c**): XMRS-1; (**d**): XMRS-1-D); (**B**) Effects of different concentrations of XL-1, XL-1-D, XMRS-1, and XMRS-1-D on the secretion of IL-6 and TNF-α in RAW264.7 cells ((**a**): XL-1; (**b**): XL-1-D; (**c**): XMRS-1; (**d**): XMRS-1-D; (**e**): XL-1; (**f**): XL-1-D; (**g**): XMRS-1; (**h**): XMRS-1-D); (**C**) Effects of different concentrations of XL-1, XL-1-D, XMRS-1, and XMRS-1-D on the relative expression of iNOS in RAW264.7 cells; Effects of different concentrations of XL-1, XL-1-D, XMRS-1, and XMRS-1-D on the relative expression of IL-6 in RAW264.7 cells ((**a**): XL-1; (**b**): XL-1-D; (**c**): XMRS-1; (**d**): XMRS-1-D) Effects of different concentrations of XL-1, XL-1-D, XMRS-1, and XMRS-1-D on the relative expression of TNF-α in RAW264.7 cells ((**a**): XL-1; (**b**): XL-1-D; (**c**): XMRS-1; (**d**): XMRS-1-D) (All data are expressed as mean ± standard deviation (mean ± SD). Differences between groups and the blank control were analyzed using one-way analysis of variance (ANOVA). Significance levels were defined as follows: * *p* < 0.05 (significant), ** *p* < 0.01 (highly significant), *** *p* < 0.001 (extremely significant), and **** *p* < 0.0001 (very highly significant)).

As shown in Figure 6A, ROS release from macrophages gradually increased with increasing concentrations of XL-1. The ROS release trend for the XL-1-D group was similar to its NO release pattern, peaking at 400 µg/mL. ROS release by the XMRS-1 and XMRS-1-D groups also showed a trend similar to their NO release, gradually increasing with fraction concentration. These results indicate that these four fractions can promote ROS release in macrophages, thereby enhancing their immune activity.

#### 2.3.5. XL-1, XL-1-D, XMRS-1, and XMRS-1-D Promoted the Expression of IL-6 and TNF-α

As depicted in Figure 6B, 1 µg/mL LPS significantly increased the expression of IL-6 and TNF-α cytokines in macrophages. Similarly, XL-1, XL-1-D, XMRS-1, and XMRS-1-D promoted the expression of IL-6 and TNF-α cytokines within the concentration range of 100 to 800 µg/mL, generally exhibiting concentration dependence. Therefore, these four XL-1, XL-1-D, XMRS-1, and XMRS-1-D can effectively promote the expression of inflammatory mediators, inducing a degree of inflammatory response in macrophages and demonstrating an immune-enhancing effect.

#### 2.3.6. Effect of XL-1, XL-1-D, XMRS-1, and XMRS-1-D on LPS-Induced mRNA Expression of iNOS, TNF-α, and IL-6 in RAW264.7 Cells

RT-qPCR results (Figure 5C) demonstrated that LPS significantly increased the mRNA expression of iNOS, TNF-α, and IL-6 in RAW264.7 cells. When the concentration of XL-1 reached 200 µg/mL, it significantly increased iNOS mRNA expression in RAW264.7 macrophages, consistent with the observed trends for NO and ROS. For XL-1-D, XMRS-1, and XMRS-1-D, iNOS mRNA expression was also significantly increased at a concentration of 400 µg/mL. At an administration concentration of 800 µg/mL, XL-1, XL-1-D, XMRS-1, and XMRS-1-D significantly increased IL-6 mRNA expression in macrophages. Similarly, under these concentration conditions, the four fractions also significantly increased TNF-α mRNA expression in macrophages.

### 2.4. Anti-Tumor Effects In Vitro

#### 2.4.1. XL-1, XL-1-D, XMRS-1, and XMRS-1-D Inhibited A549 Cells Proliferation

**Figure 7:**
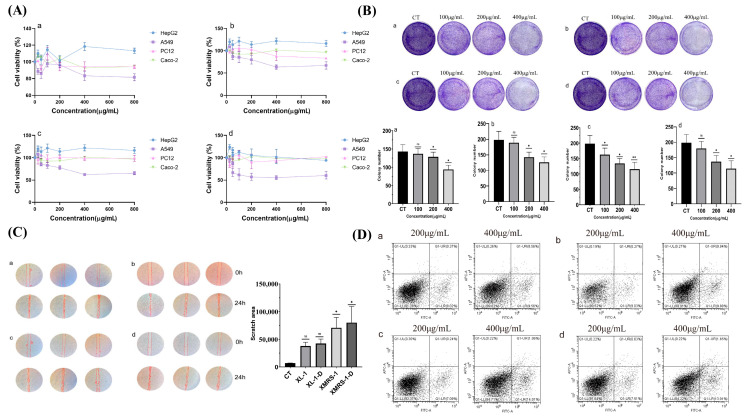
(**A**) Effects of different concentrations of XL-1, XL-1-D, XMRS-1, and XMRS-1-D on A549 cell viability ((**a**): XL-1; (**b**): XL-1-D; (**c**): XMRS-1; (**d**): XMRS-1-D); (**B**) Effects of different concentrations of XL-1, XL-1-D, XMRS-1, and XMRS-1-D on the colony formation of A549 cells ((**a**): XL-1; (**b**): XL-1-D; (**c**): XMRS-1; (**d**): XMRS-1-D); (**C**) Effects of 400 μg/mL XL-1, XL-1-D, XMRS-1, and XMRS-1-D of each component on the migration ability of A549 cells ((**a**): XL-1; (**b**): XL-1-D; (**c**): XMRS-1; (**d**): XMRS-1-D); (**D**): Effects of different concentrations of XL-1, XL-1-D, XMRS-1, and XMRS-1-D of A549 cells ((**a**): XL-1; (**b**): XL-1-D; (**c**): XMRS-1; (**d**): XMRS-1-D). (All data are expressed as mean ± standard deviation (mean ± SD). Differences between groups and the blank control were analyzed using one-way analysis of variance (ANOVA). Significance levels were defined as follows: * *p* < 0.05 (significant), ** *p* < 0.01 (highly significant)).

The cytotoxicity of XL-1, XL-1-D, XMRS-1, and XMRS-1-D were assessed against A549, PC12, HepG2, and Caco-2 cells co-cultured with different fractions concentrations for 48 h. Cell viability was determined using the CCK-8 method (Figure 7A). In the concentration range of 0 to 800 µg/mL, XL-1, XL-1-D, XMRS-1, and XMRS-1-D derived from hadal bacterial strains did not show an inhibitory effect on HepG2 cells. For PC12 and Caco-2 cells, some inhibitory effect was observed within the 0 to 800 µg/mL range. A significant inhibitory effect was shown against A549 cells. Notably, when the fraction concentration reached 400 µg/mL, the inhibitory effect on A549 cells was optimal; further increases in concentration did not lead to a corresponding increase in inhibition. These results indicated that XL-1, XL-1-D, XMRS-1, and XMRS-1-D have a certain selectivity for the inhibitory activity against these tumor cells.

#### 2.4.2. XL-1, XL-1-D, XMRS-1, and XMRS-1-D Inhibited the Colony Formation Ability of A549 Cells

Plate cloning experiments (Figure 7B) showed that, as the concentrations of XL-1, XL-1-D, XMRS-1, and XMRS-1-D increased, the density of colony formation significantly decreased. The number of colonies (Figure 6B, visually) was statistically analyzed using ImageJ software (1.8.0_322). For XL-1, XL-1-D, and XMRS-1-D, the number of colonies began to decrease significantly at 200 µg/mL (*p* < 0.05). For XMRS-1, the number of colonies was significantly reduced at 200 µg/mL (*p* < 0.05), with a more pronounced decrease at 400 µg/mL (*p* < 0.01). These experimental results indicated that XL-1, XL-1-D, XMRS-1, and XMRS-1-D could inhibit the in vitro tumorigenic potential of A549 cells to a certain extent.

#### 2.4.3. XL-1, XL-1-D, XMRS-1, and XMRS-1-D Inhibited the Migration Ability of A549 Cells

After A549 cells were treated with XL-1, XL-1-D, XMRS-1, and XMRS-1-D components for 24 h, images were processed using ImageJ software(1.8.0_322), and the scratch area was marked (Figure 7C). The results showed that XL-1, XL-1-D, XMRS-1, and XMRS-1-D could inhibit the migration ability of A549 cells to some extent, with XMRS-1 exhibiting the strongest inhibitory effect. The scratch area was calculated by ImageJ (Figure 7C). After 48 h of incubation, the scratch areas for XMRS-1 and XMRS-1-D treated cells were significantly larger than that of the blank control group (*p* < 0.05).

#### 2.4.4. XL-1, XL-1-D, XMRS-1, and XMRS-1-D Induced Apoptosis in A549 Cells

After 48 h of treatment with XL-1, XL-1-D, XMRS-1, and XMRS-1-D, A549 cells exhibited induced apoptosis in a somewhat concentration-dependent manner (Figure 7D). The number of cells in the Q1 quadrant (mechanical death/necrotic cells) was minimal, indicating good cell condition and reliable experimental data. Specifically, for XL-1, as the concentration increased from 200 µg/mL to 400 µg/mL, the combined proportion of early and late apoptotic cells increased from 8.02% to 9.56%. For XL-1-D, an increase in concentration from 200 µg/mL to 400 µg/mL led to an increase in early and late apoptotic cells from 9.03% to 9.88%. For XMRS-1, increasing the concentration from 200 µg/mL to 400 µg/mL resulted in an increase in early and late apoptotic cells from 7.09% to 14.01%. For XMRS-1-D, as the concentration rose from 200 µg/mL to 400 µg/mL, the proportion of early and late apoptotic cells increased from 7.51% to 13.91%.

#### 2.4.5. Effect of XMRS-1 and XMRS-1-D on Apoptosis-Related Gene Expression in A549 Cells

**Figure 8:**
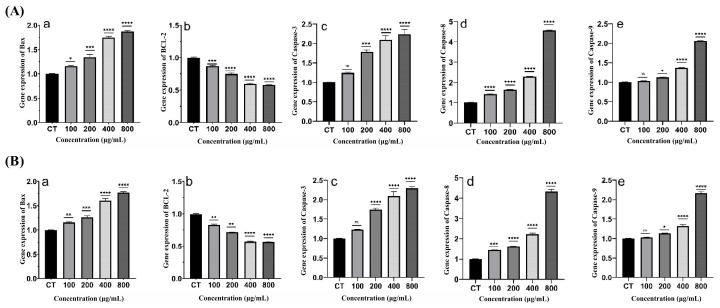
(**A**): Effects of different concentrations of XMRS-1 on the expression of apoptosis-related genes in A549 cells ((**a**): Bax; (**b**): BCL-2; (**c**): Caspase-3; (**d**): Caspase-8; (**e**): Caspase-9). (**B**): Effects of different concentrations of XMRS-1-D on the expression of apoptosis-related genes in A549 cells ((**a**): Bax; (**b**): BCL-2; (**c**): Caspase-3; (**d**): Caspase-8; (**e**): Caspase-9). (All data are expressed as mean ± standard deviation (mean ± SD). Differences between groups and the blank control were analyzed using one-way analysis of variance (ANOVA). Significance levels were defined as follows: * *p* < 0.05 (significant), ** *p* < 0.01 (highly significant), *** *p* < 0.001 (extremely significant), and **** *p* < 0.0001 (very highly significant)).

The expression of apoptosis-related genes in A549 cells was detected by qPCR after 48 h of treatment. No significant differences in the expression of tested apoptosis-related genes were found after treatment with different concentrations of XL-1 and XL-1-D. However, as shown in Figure 8, after treatment with XMRS-1 and XMRS-1-D for 48 h, the expression levels of pro-apoptotic genes Bax, Caspase-3, Caspase-8, and Caspase-9 were significantly increased (*p* < 0.05, *p* < 0.01, *p* < 0.001, and *p* < 0.0001). This increase was positively correlated with the concentration of XMRS-1 and XMRS-1-D, indicating concentration-dependent apoptosis induction. Furthermore, the relative expression of the anti-apoptotic gene Bcl-2 decreased significantly with increasing XMRS-1 and XMRS-1-D concentration (*p* < 0.001).

## 3. Discussions

Natural polysaccharides are an important treasure trove for discovering new drug-derived molecules [40,41,42,43,44,45]. Globally, discovering natural products with novel structures and unique biological activities from extreme environmental microorganisms, such as hadal microorganisms, has become an important strategy for discovering innovative drugs [41,42,43,44,45,46,47,48,49,50,51]. In this study, XL-1, XMRS-1, XL-1-D, and XMRS-1-D were characterized by chemical composition and their antitumor and immunological activities evaluated in vitro. These results showed that there was significant diversity in their monosaccharide composition and structural properties. The initial extract XL-1 and XMRS-1 had a more complex monosaccharide composition compared with further purified fractions XL-1-D and XMRS-1-D, which were mainly composed of mannose. This suggested the complexity and diversity of the structure of these polysaccharides. Even within the same species, there are multiple different structures of polysaccharides. In general, there is a close correlation between structure and activity, where structural disparities indicate differences in activity. It is essential to choose the appropriate purification method [52,53,54]. Here, the immunological activity and anti-tumor activity of the four fractions were evaluated. In terms of immunomodulatory activity and potential, firstly, XL-1, XL-1-D, XMRS-1, and XMRS-1-D had no obvious toxicity to RAW264.7 cellsat concentrations up to 1000 μg/mL and could promote cell proliferation, showing good biocompatibility. Further studies have shown that XL-1, XL-1-D, XMRS-1, and XMRS-1-D can effectively activate macrophages, specifically enhancing phagocytic ability, promoting the production of key effector molecules NO and ROS, and upregulating the expression of important proinflammatory cytokines IL-6 and TNF-α. These findings together confirm that XL-1, XL-1-D, XMRS-1, and XMRS-1-D have the potential to activate macrophages and enhance innate and adaptive immune responses, supporting their development prospects as immunopotentiators or vaccine adjuvants. Comparing these activities with the previous chemical structures, the differences in activation intensity and dose–response of different components suggest the existence of structure–activity relationships, indicating that specific chemical structures are crucial for their immunomodulatory functions. In addition to immunomodulatory activity, this study further explored the potential of XL-1, XL-1-D, XMRS-1, and XMRS-1-D to inhibit tumor cell growth directly. Preliminary screening showed that XL-1, XL-1-D, XMRS-1, and XMRS-1-D showed the highest relative cytotoxicity against human non-small cell lung cancer cells A549, which was superior to the effects on PC12, HepG2, and Caco-2 cells, indicating that they may have certain tumor cell targeting or sensitivity differences. Based on this, we used A549 cells as a model for in-depth research. The results showed that XL-1, XL-1-D, XMRS-1 and XMRS-1-D could significantly inhibit the cloning ability and cell migration ability of A549 cells, and the inhibitory effect on migration was concentration-dependent. To explore its mechanism of action, we detected by flow cytometry that within the concentration range of 200–400 μg/mL, XL-1, XL-1-D, XMRS-1, and XMRS-1-D could induce apoptosis in A549 cells in a concentration-dependent manner, among which XMRS-1 and XMRS-1-D had more significant effects on inducing apoptosis [55,56,57]. Notably, although these four mannan-containing polymers contain relatively low protein contents, their biological activities are presumed to originate from the synergistic effects between the polysaccharide backbone and covalently bound proteins, and this mechanism warrants further investigation for elucidation.

It is worth noting that mannan-containing polymers from strain XMRS204 not only show good immune activation ability but also perform particularly well in anti-tumor activity. This suggests that these fractions from strain XMRS204 have a chemical structure conducive to dual antitumor effects. In contrast, although the more complex fraction XL-1 from strain XL111 also has immune and antitumor activities, its direct antitumor efficacy seems to be inferior to that of fractions from strain XMRS204. This preliminary structure–activity relationship (SAR) is extremely valuable, suggesting that specific monosaccharide composition, molecular weight range, or conformational characteristics may determine its main biological activity spectrum separately or jointly [58,59,60]. Besides, they had potential anticancer activities, partially due to inhibiting colony formation and migrating of A549 Cells, plus inducing apoptosis of A549 Cells.

Much in-depth research work is still needed in the future, including exploring the structure–activity relationship of these extracellular polysaccharides in anti-tumor effects, further clarifying their structural characteristics, and investigating their anti-tumor mechanism and targets.

## 4. Materials and Methods

### 4.1. Strains and Experimental Consumables

Hadal bacteria *P. pulmonis* XL111 and *P. pulmonis* XMRS204 were donated by Professor Yu Xi from the College of Oceanography and Ecological Science, Shanghai Ocean University. Experimental materials: Sodium hydroxide, sulfuric acid, sodium chloride, phenol, anhydrous ethanol, hydrochloric acid, phenol, borax, carbazole, trichloroacetic acid, trifluoroacetic acid, and chloroform were purchased from Sinopharm Chemical Reagent Co., Ltd. (Shanghai, China); 1-phenyl-3-methyl-5-pyrazolone (PMP) was purchased from Shanghai Yuanye Bio-Technology Co., Ltd. (Shanghai, China); L-rhamnose, L-fucose, L-arabinose, D-xylose, D-mannose, D-galactose, D-glucose, and galacturonic acid standards were purchased from Fluka (Shanghai, China); DEAE Sepharose Fast Flow and High Resolution were produced by Cytiva Bio-technology Co., Ltd. (Hangzhou, China); RAW264.7: mouse mononuclear macrophages, DMEM high-glucose medium, and disposable 96-well plates were purchased from Wuhan Servicebio Technology Co., Ltd. (Wuhan, China); phosphate buffered saline (PBS), Cell Counting Kit-8 (CCK-8), Lipopolysaccharide (LPS) was purchased from Sigma-Aldrich (Shanghai, China), and trypsin digestion solution (containing EDTA) were purchased from Shanghai Titan Technology Co., Ltd. (Shanghai, China); BCA kit, DCFHDA, neutral red staining solution, NO kit, cell lysis solution, and RIPA cell lysis solution were purchased from Beyotime Biotechnology Co., Ltd. (Shanghai, China); mouse interleukin (IL-6) kit and mouse tumor necrosis factor (TNF-α) kit were purchased from Jiangsu Jingmei Biotechnology Co., Ltd. (Jiangsu, China); SPARKeasy Cell RNA Kit cell RNA rapid extraction kit was purchased from Shandong Sparkjade Biotechnology Co., Ltd. (Shandong, China); Evo M-MLV reverse transcription kit, SYBR Green Pro Taq HS premixed qPCR kit, and Evo M-MLV reverse transcription kit were purchased from Hunan Accurate Biotechnology Co., Ltd. (Hunan, China); human non-small cell lung cancer cells (A549), rat adrenal pheochromocytoma cells (PC12), human hepatoma cells (HepG2), and human colorectal adenocarcinoma cells (Caco-2) were purchased from the Cell Bank of the Chinese Academy of Sciences; crystal violet was purchased from Yonghua Chemical Co., Ltd. (Jiangsu, China); all reagents used in our studies were of analytical grade.

### 4.2. Strain Isolation and Purification

Two *Psychrobacter* strains were isolated from the gut contents of hadal amphipods (*Hirondellea gigas*) collected at depths exceeding 10,000 m in the Mariana Trench. *Psychrobacter pulmonis* XL111 was obtained through Luria-Bertani (LB) agar medium, whereas *Psychrobacter pulmonis* XMRS204 was isolated using De Man, Rogosa, and Sharpe (MRS) agar medium. The isolation procedures were conducted following the protocol described in our previous methodology [61].

#### 4.2.1. Colony Characteristics

When cultivated on LB agar for 72 h, both strains formed off-white colonies exhibiting smooth surfaces with moist textures, featuring semi-transparent edges, regular contours, and a raised center. Notably, *P. pulmonis* XL111 demonstrated non-adherent behavior, in contrast to the distinctively adhesive phenotype observed in *P. pulmonis* XMRS204 under identical culture conditions.

#### 4.2.2. Large-Scale Cultivation and Crude Fractions Extraction

*P. pulmonis* XL111 and *P. pulmonis* XMRS204 were cultured on LB agar medium for 3 days. Individual colonies were picked and inoculated into 200 mL liquid LB medium until reaching mid-exponential phase (OD600 ≈ 0.5). Subsequently, 1% (*v*/*v*) inoculum were transferred into 20 L sterile LB medium for scaled-up cultivation (72 h, 28 °C, 180 rpm). Afterward, the culture was centrifuged at 10,000 rpm for 40 min at 4 °C, and the supernatant was collected for polysaccharides extraction.

#### 4.2.3. Preparation of Extracellular Fractions from Marine Microorganisms

Take the culture supernatant of the bacterium *Psychrobacter* in the hadal environment, concentrate it with a rotary evaporator, and precipitate it with ethanol in the corresponding proportion at 4 °C overnight. After the precipitation is completed, centrifuge, discard the supernatant, re-dissolve the precipitate, and freeze-dry to obtain crude extracellular fractions. During the fractions’ purification process, the protein content often affects the yield of fractions in the later stage and the purity of fractions. The Savage method is often used to remove proteins [62]. In this paper, dissolve the crude fractions in deionized water, prepare 100 mL of 30% trichloroacetic acid solution, and mix the two solutions to make the final trichloroacetic acid concentration reach 15%. Stir in an ice bath for 4 h. After the end, centrifuge, remove the precipitate, and keep the supernatant. The supernatant is adjusted to neutral with 10% NaOH, and then dialyzed using a dialysis bag with a molecular weight of 3500 Da for three days. Next, the inner liquid is taken out, concentrated and dried to obtain these fractions. Meanwhile, the sterile yeast culture medium also underwent the same ethanol precipitation and deproteinization procedures.

Take the samples, dissolve in deionized water, centrifuge, and take the supernatant. Load the supernatant into a DEAE cellulose anion column, and use different concentrations of sodium chloride solution for gradient elution, with a sodium chloride concentration of (0.1, 0.2, 0.3, 0.4, 0.5 mol/L). For sulfuric acid-phenol detection, collect and combine the 0.1 M NaCl eluate, concentrate, dialyze, and freeze-dry to obtain marine microbial extracellular fractions XL-1 and XMRS-1. Use Sephadex S-100 gel column for separation and purification to obtain marine microbial extracellular fractions XL-1-D and XMRS-1-D.

### 4.3. Analysis of the Physicochemical Properties of the Four Fractions

The total sugar determination method is the phenol-sulfuric acid method, and the standard is mannose [63]. The content of protein was determined by Bicinchoninic Acid (BCA) protein Assay kit. The uronic acid content was determined by the carbazole sulfate method, with galactose whole acid as the standard [64].

#### 4.3.1. Determination of Molecular Weight

The HPGPC method was used to detect sample purity and molecular weight. Weigh 5 mg of XL-1-D and XMRS-1-D samples respectively, dissolve in 1 mL of ultrapure water, prepare 5 mg/mL sample solution, filter through 0.22 μm aqueous microporous membrane, and use HPLC-RI (Model 2414, Waters Corporation, Milford, MA, USA) to analyze the sample molecular weight distribution. In the experiment, chromatographic columns TOSOH TSK PWXL G4000 and TSK PWXL G2500 were selected in series, the mobile phase was 0.15 mol/L NaNO_3_, the flow rate was 0.5 mL/min, and the column temperature was 35 °C. Empower software 3 analyzed the data and calculated the molecular weight.

#### 4.3.2. Determination of Monosaccharide Composition

The monosaccharide composition of XL-0, XL-1, XMRS-0, XMRS-1, XL-1-D, and XMRS-1-D was determined by the PMP pre-column derivatization method [65]. Briefly, the fractions samples were completely hydrolyzed with TFA. The excess TFA was removed by rotary evaporation. Next, PMP derivatization was performed. The derivatized samples were analyzed by HPLC. Analysis conditions: the chromatographic column was an XDB-C18 column; the mobile phase was 0.1 mol/L phosphate (pH 6.7) buffer-acetonitrile (volume ratio of 83:17); the column temperature was 30 °C, the detection wavelength was 254 nm, the flow rate was 1 mL/min, and the injection volume was 20 μL [66].

#### 4.3.3. Fourier Transform Infrared Spectroscopy

Take an appropriate amount of XL-1, XMRS-1, XL-1-D, and XMRS-1-D fractions samples that have been pre-dried to a constant weight, and the sample volume is enough to cover the small hole of the Fourier infrared spectrometer. The number of scans is 16 times, and the scanning range is 400–4000 cm^−1^.

#### 4.3.4. Morphological Characterization of Fractions

Weigh a certain amount of freeze-dried XL-1, XL-1-D, XMRS-1 and XMRS-1-D samples and plate them with metal films. Scan the electron microscope to observe the microstructure of the fractions and the morphological characteristics of the fractions’ samples at different magnifications.

#### 4.3.5. Amino Acid Composition of Purified Fractions

Weigh XL-1-D and XMRS-1-D 5 mg, dissolve in 5 mL 6 mol/L HCl, put into hydrolysis tube, isolate oxygen, and hydrolyze in 110 °C ± 2 °C electric heating constant temperature box for 22 h. Cool to room temperature, filter the hydrolyzate with 0.22 μm filter membrane, and transfer all the liquid into 25 mL volumetric flask to make up the volume. Dry it in a vacuum drying oven several times, and finally add sodium citrate buffer solution (pH = 2.2), shake well, filter through 0.22 μm, and detect on the machine.

#### 4.3.6. NMR Analysis

For NMR analysis, samples were dehydrated, exchanged and dissolved in 0.5 mL D2O under vacuum. Using acetone as the internal standard, its ^1^H and ^13^C NMR spectra were measured at room temperature. NMR spectra were recorded using a Varian Mercury 600 NMR spectrometer [67].

### 4.4. Immunomodulatory Effects of Fractions

#### 4.4.1. Effect of XL-1, XL-1-D, XMRS-1, and XMRS-1-D on the Viability and Proliferation of RAW264.7 Cells

Cell type: mouse mononuclear macrophages (RAW264.7). Growth conditions: DMEN high-glucose medium with 10% FBS, the medium changed once a day. Incubator 37 °C, 5% carbon dioxide. Cells should be cultured to the logarithmic phase, then gently blown down and evenly distributed on a 96-well plate.

CCK-8 experimental steps: The cells were divided into 9 groups, including 1 blank control group (containing culture medium only) and 8 experimental groups (containing 0 µg/mL, 25 µg/mL, 50 µg/mL, 100 µg/mL, 200 µg/mL, 400 µg/mL, 800 µg/mL, 1000 µg/mL), and each group was paralleled 3 times. 100 μL complete culture medium was added to each well of a 96-well plate to culture 1 × 104 cells. After 24 h of culture, the old culture medium was removed, gently washed with PBS, 100 μL of culture medium containing different concentrations of fraction solution was added, and the blank group was not added. After continuing to culture for 48 h, the old culture medium was discarded, protected from light, and 100 μL of culture medium containing 10% CCK8 was added to each well, and the absorbance was measured at 450 nm.

#### 4.4.2. Effects on Macrophage Phagocytic Function

The experiment was divided into 5 groups: 1 blank group (containing culture medium only), 1 control group (containing 1 µg/mL LPS), and 5 experimental groups (containing 50 µg/mL, 100 µg/mL, 200 µg/mL, 400 µg/mL, and 800 µg/mL solution), with 3 replicates in each group. 1 × 104 cells were cultured in 100 μL complete culture medium per well of a 96-well plate. After 24 h of culture, the old culture medium was discarded, the plates were gently washed once with PBS, and then 0.01% neutral red solution at 37 °C was added for incubation for 2 h. The neutral red dye was then gently aspirated, the plates were washed three times with PBS, 200 lysis solution was added to each well and, after 2 h of incubation, the absorbance was measured at 450 nm using a multifunctional microplate reader.

#### 4.4.3. Effect on Nitric Oxide (NO) Production by Macrophages

The experiment was divided into 5 groups: 1 blank group (containing only culture medium), 1 control group (containing 1 µg/mL LPS), and 5 experimental groups (containing 50 µg/mL, 100 µg/mL, 200 µg/mL, 400 µg/mL, 800 µg/mL solution), each group repeated 3 times. An amount of 100 μL complete culture medium was added to each well of a 96-well plate to culture 2 × 104 cells. After 48 h of culture, the culture medium was removed, centrifuged at 10,000 rpm and 4 °C for 3 min, and the supernatant was taken for later use. Then, 50 µL of sample was taken from each well of the 96-well plate, 50 µL of Griess reagent I was first added, and then 50 µL of Griess reagent II was added, mixed at room temperature and a wait occurred for 5 min for reaction. Finally, the absorbance was measured at 540 nm using an ELISA reader.

#### 4.4.4. Effect on Reactive Oxygen Species (ROS) Production by Macrophages

The experiment was divided into 5 groups: 1 blank group (containing culture medium only), 1 control group (containing 1 µg/mL LPS), and 5 experimental groups (containing 50 µg/mL, 100 µg/mL, 200 µg/mL, 400 µg/mL, 800 µg/mL solution), each group was paralleled 3 times. 100 μL complete culture medium was added to each well of a 96-well plate to culture 2 × 104 cells. After 48 h of culture, 100 µL of culture medium containing 10 μmol/mL DCFHDA was added under light-proof conditions and incubated at 37 °C for 20 min. Then the cells were washed 3 times with serum-free culture medium, 50 μL PBS was added, and photos were taken under a fluorescence microscope.

#### 4.4.5. Effect on IL-6 and TNF-α Expression by Macrophages

The mouse interleukin (IL-6) kit and mouse tumor necrosis factor (TNF-α) kit were used to detect the content of cell secretion factors IL-6 and TNF-α. Take the cell supernatant in Section 4.2.3 for later use, follow the instructions of the kit, and measure the absorbance at 450 nm with a microplate reader.

#### 4.4.6. LPS Induces mRNA Expression of iNOS, TNF-α, and IL-6 in RAW264.7 Cells

The cells were divided into 5 groups: 1 blank group (containing only culture medium), 1 control group (containing 1 µg/mL LPS), and 3 experimental groups (containing 100 µg/mL, 200 µg/mL, and 400 µg/mL solutions), with 3 parallel samples in each group.

When the cells grew to the logarithmic phase, they were plated with 3 × 106 cells per well. After overnight adherence, the cells were gently washed and culture medium containing 100 µg/mL, 200 µg/mL, and 400 µg/mL solutions was added and incubated for 48 h. After that, RNA was extracted according to the Sparkeasy Cell RNA Kit of Sparkeasy.

After measuring the concentration of the obtained sample, reverse transcription is performed. Data analysis is performed to determine whether the amplification data is available based on the melting curve, and the expression level can be determined by the ready-to-use 2-∆∆Ct value.

The primers used are listed in the following table (Table 3).

### 4.5. Anti-Tumor Effects

#### 4.5.1. Assessment of Cytotoxicity Against Various Tumor Cell Lines

Cell types: human non-small cell lung cancer cells (A549), rat adrenal pheochromocytoma cells (PC12), human hepatoma cells (HepG2), human colorectal adenocarcinoma cells (Caco-2).

Growth conditions: DMEM high glucose medium (10% FBS), medium change every 2 days, incubator at 37 °C, 5% carbon dioxide concentration. Culture to logarithmic phase, trypsin digestion, and plate.

CCK-8 experiment: cells were divided into 9 groups, specifically divided into: 1 blank group (containing culture medium only), 8 experimental groups (containing 0 µg/mL, 25 µg/mL, 50 µg/mL, 100 µg/mL, 200 µg/mL, 400 µg/mL, 800 µg/mL, 1000 µg/mL), 3 parallels in each group. The cell density was 1 × 105, and the cells were evenly distributed in a 96-well plate. 100 µL of culture medium was added to each well. After 24 h of culture, the old culture medium was discarded, and 100 µL of culture medium containing different concentrations of solution was added to each well. The blank group was added with culture medium without fractions. After 48 h of culture, the old culture medium was discarded. 100 µL of culture medium containing 10% CCK-8 was added, incubated in the dark for 2 h, and the absorbance was measured at 450 nm.

#### 4.5.2. Effect on the Colony Formation Ability of A549 Cells

The cells in the logarithmic phase were trypsinized, and 5000 cells were plated in each well until the cells formed a colony before drug administration. The experiment was divided into a blank group and three experimental groups (containing 100 µg/mL, 200 µg/mL and 400 µg/mL of drugs), and each group had three parallel treatments. The culture medium was replaced every three days. After 12 days of culture, the old culture medium was removed, washed with PBS, and then fixed with 4% paraformaldehyde at room temperature for 1 h. Then use 1% crystal violet to stain for 15 min, and then gently wash with tap water. Finally, use Image J software (1.8.0_322) for photo recording and calculation analysis.

#### 4.5.3. Effect on the Migration Ability of A549 Cells

Select logarithmic phase cells for trypsin digestion, and evenly spread 5 × 105 A549 cells in a 6-well plate. Draw three parallel lines on the bottom back of the 6-well plate, and the cultured cells reach 80% confluence. At this time, scratch with a 200 μL pipette tip, wash with PBS, and record the 0 h state. Next, add culture medium containing different concentrations of solution and co-culture with A549 cells for 48 h. The specific experimental grouping includes a blank group and three experimental groups (containing 100 µg/mL, 200 µg/mL and 400 µg/mL solutions), each group has three parallel treatments. After 48 h of culture, take photos and record.

#### 4.5.4. Effect on Apoptosis in A549 Cells

After culturing the cells to the logarithmic phase, remove the six-well plate and add 3 × 105 A549 cells to each well. Culture the cells for 6–8 h until the cells adhere to the wall. The experimental groups were set to 200 μg/mL and 400 μg/mL, and the culture medium containing solution was added and cultured for 48 h. Collect the cells, centrifuge at 300 g for 5 min, and discard the supernatant. Wash the cells once with PBS, add 100 μL of diluted 10× Annexin V Binding Buffer to resuspend the cells. Add 2.5 μL of Annexin V-FITC Reagent and 2.5 μL of PI Reagent, incubate at room temperature in the dark for 20 min, and then add 400 μL of diluted 10× Annexin V Binding Buffer. Immediately test on the machine.

#### 4.5.5. Effect on Apoptosis-Related Gene Expression in A549 Cells

Cultivate the cells according to Section 4.5.1. The extraction and detection steps are the same as Section 4.4.6.

After measuring the concentration of the obtained sample, reverse transcription is performed, that is, the reaction system containing template RNA, primers, Reaction Buffer, RNase Inhibitor, dNTP Mix, and M-MLV reverse transcriptase is placed in the PCR instrument. Set the reverse transcription program and perform reverse transcription. Take an appropriate amount of reverse transcription solution, add 2× QuantiNova SYBR Green PCR Master Mix, front primers, and back primers, use a 20 μL system, and set the program fluorescence quantitative instrument for amplification. Finally, data analysis is performed to determine whether the amplification data is available based on the melting curve, and the expression level can be determined by the ready-to-use 2^−∆∆Ct^ value.

The primers used are as follows (Table 4).

### 4.6. Statistical Analysis

This experiment used Excel, Graphpad Pism9.5, OriginPro 8.5.1, etc., for data processing, and the final measurement results were expressed as mean ± standard error of the mean (Mean ± SEM). All experiments were repeated 3 times. Statistical significance of each group: ns means no significant difference; * *p* < 0.05 means significant difference; ** *p* < 0.01 means some significant difference, *** *p* < 0.001 means extremely significant difference, **** *p* < 0.0001 means very significant difference.

## 5. Conclusions

In summary, this study revealed that the four mannan-containing polymers from hadal microorganism have significant immunomodulatory and anti-tumor biological activities by integrating multidisciplinary cross-studies, such as chemistry, immunology, and tumor biology, and preliminarily established the relationship between structure and activity, revealing that XL-1, XL-1-D, XMRS-1, and XMRS-1-D from hadal bacteria, especially XMRS-1 and XMRS-1-D, show great development potential as new anticancer drugs. These research results not only enrich our understanding of the medicinal value of natural products from extreme environmental microorganisms, but more importantly, provide highly potential candidate molecules and important theoretical basis for the development of new anti-tumor molecules or immunotherapy adjuvants with novel mechanisms of action.

## Figures and Tables

**Table 1:** Analysis of the physicochemical properties and monosaccharide composition of XL-1, XL-1-D, XMRS-1, and XMRS-1-D.

Sample Name	Total Sugar Content	Protein Content	Uronic Acid Content	Monosaccharide Composition
				Man:GlcN:GlcA:GalN:Gal:Glc
XL-1	43.56%	17.31%	3.65%	35.45:7.28:14.66:36.80:2.84:0
XL-1-D	66.82%	10.02%	5.14%	89.41:4.36:0:0:0:6.22
XMRS-1	37.54%	13.99%	5.27%	56.42:1.97:0:0:0:41.61
XMRS-1-D	49.80%	12.30%	5.60%	93.26:1.96:0:0:1.04:3.74

**Table 2:** Amino acid composition of XL-1-D and XMRS-1-D (residues/1000 residues).

Sample Name	Amino Acid Species														
	Asp	Thr	Ser	Glu	Gly	Ala	Val	Ile	Leu	Tyr	Phe	Lys	His	Arg	Pro
XL-1-D	82.35 ± 2.20	23.17 ± 0.61	42.86 ± 1.16	104.09 ± 2.78	389.13 ± 10.20	123.62 ± 3.34	18.54 ± 4.50	7.16 ± 0.23	14.41 ± 0.42	1.90 ± 0.07	5.55 ± 0.16	23.13 ± 0.62	2.16 ± 0.06	24.06 ± 0.65	137.88 ± 3.65
XMRS-1-D	88.36 ± 0.96	25.04 ± 0.27	42.19 ± 0.46	106.15 ± 1.16	383.70 ± 1.09	124.58 ± 1.39	1.89 ± 0.02	10.13 ± 0.11	22.95 ± 0.25	3.47 ± 0.04	8.26 ± 0.09	25.65 ± 0.28	3.28 ± 0.03	17.44 ± 0.19	136.88 ± 3.65

**Table 3:** Primers used for RT-PCR analysis.

	Upstream Primer	Downstream Primer
GAPDH	AATGGATTTGGACGCATTGGT	TTTGCACTGGTACGTGTTGAT
iNOS	CTCTTCGACGACCCAGAAAAC	CAAGGCCATGAAGTGAGGCTT
TNF-α	CAGGTTCTCTTCAAGGGACAAGGC	TGACGGCAGAGAGGAGGTTGAC
IL-6	CTTCTTGGGACTGATGCTGGTGAC	TCTGTTGGGAGTGGTATCCTCTGTG

**Table 4:** Primers of Apoptosis-Related Gene for RT-PCR analysis.

	Upstream Primer	Downstream Primer
GAPDH	TGTGGGCATCAATGGATTTGG	ACACCATGTATTCCGGGTCAAT
Bax	CCCGAGAGGTCTTTTTCCGAG	CCAGCCCATGATGGTTCTGAT
Caspase-3	CATGGAAGCGAATCAATGGACT	CTGTACCAGACCGAGATGTCA
Caspase-8	TTTCTGCCTACAGGGTCATGC	GCTGCTTCTCTCTTTGCTGAA
Caspase-9	CTCAGACCAGAGATTCGCAAAC	GCATTTCCCCTCAAACTCTCAA
BCL-2	GGTGGGGTCATGTGTGTGG	CGGTTCAGGTACTCAGTCATCC

## Data Availability

The data presented in this study are available on request from the corresponding author.
